# Unveiling the Mechanisms of a Remission in Major Depressive Disorder (MDD)-like Syndrome: The Role of Hippocampal Palmitoyltransferase Expression and Stress Susceptibility

**DOI:** 10.3390/biom15010067

**Published:** 2025-01-05

**Authors:** Careen A. Schroeter, Anna Gorlova, Michael Sicker, Aleksei Umriukhin, Alisa Burova, Boris Shulgin, Sergey Morozov, Joao P. Costa-Nunes, Tatyana Strekalova

**Affiliations:** 1 Rehabilitation Research Unit, Preventive and Environmental Medicine, Kastanienhof Clinic, Statthalterhofweg, 50858 Cologne-Junkersdorf, Germany; 2 FGBNU, Institute of General Pathology and Pathophysiology, Russian Academy of Medical Sciences, 125315 Moscow, Russia; anna.gorlova204@gmail.com (A.G.); aliceinmsk@gmail.com (A.B.);; 3 Research and Education Resource Center, Peoples Friendship University of Russia (RUDN University), 117198 Moscow, Russia; 4 Department of Normal Physiology and Department of Mathematics, Mechanics and Mathematical Modeling, Institute of Computer Science and Mathematical Modeling, Sechenov First Moscow State Medical University, 119991 Moscow, Russiabshulgin83@gmail.com (B.S.); 5 Laboratory of Engineering Profile Physical and Chemical Methods of Analysis, Korkyt Ata Kyzylorda State University, Kyzylorda 120014, Kazakhstan; 6 Faculdade de Medicina, Universidade de Lisboa, Campo Grande, 1649-028 Lisboa, Portugal; jpcosta.nunes@gmail.com; 7 Division of Molecular Psychiatry, Center of Mental Health, University of Würzburg, 97080 Wuerzburg, Germany

**Keywords:** posttranslational modifications, DHHC8, palmitoylation, hippocampus, stress-induced anhedonia, antidepressants, mouse

## Abstract

Post-translational modifications of proteins via palmitoylation, a thioester linkage of a 16-carbon fatty acid to a cysteine residue, reversibly increases their affinity for cholesterol-rich lipid rafts in membranes, changing their function. Little is known about how altered palmitoylation affects function at the systemic level and contributes to CNS pathology. However, recent studies suggested a role for the downregulation of palmitoyl acetyltransferase (DHHC) 21 gene expression in the development of Major Depressive Disorder (MDD)-like syndrome. Here, we sought to investigate how susceptibility (sucrose preference below 65%) or resilience (sucrose preference > 65%) to stress-induced anhedonia affects DHHC gene expression in the hippocampus of C57BL/6J mice during the phase of spontaneous recovery from anhedonia. Because MDD is a recurrent disorder, it is important to understand the molecular mechanisms underlying not only the symptomatic phase of the disease but also a state of temporary remission. Indeed, molecular changes associated with the application of pharmacotherapy at the remission stage are currently not well understood. Therefore, we used a mouse model of chronic stress to address these questions. The stress protocol consisted of rat exposure, social defeat, restraint stress, and tail suspension. Mice from the stress group were not treated, received imipramine via drinking water (7 mg/kg/day), or received intraperitoneal injections of dicholine succinate (DS; 25 mg/kg/day) starting 7 days prior to stress and continuing during a 14-day stress procedure. Controls were either untreated or treated with either of the two drugs. At the 1st after-stress week, sucrose preference, forced swim, novel cage, and fear-conditioning tests were carried out; the sucrose test and 5-day Morris water maze test followed by a sacrifice of mice on post-stress day 31 for all mice were performed. Transcriptome Illumina analysis of hippocampi was carried out. Using the RT-PCR, the hippocampal gene expression of *Dhhc3*, *Dhhc7*, *Dhhc8*, *Dhhc13*, *Dhhc14*, and *Dhhc21* was studied. We found that chronic stress lowered sucrose preference in a subgroup of mice that also exhibited prolonged floating behavior, behavioral invigoration, and impaired contextual fear conditioning, while auditory conditioning was unaltered. At the remission phase, no changes in the sucrose test were found, and the acquisition of the Morris water maze was unchanged in all groups. In anhedonic, but not resilient animals, *Dhhc8* expression was lowered, and the expression of *Dhhc14* was increased. Antidepressant treatment with either drug partially preserved gene expression changes and behavioral abnormalities. Our data suggest that *Dhhc8* and *Dhhc14* are likely to be implicated in the mechanisms of depression at the remission stage, serving as targets for preventive therapy.

## 1. Introduction

The identification of biological determinants of individual dynamics of the course of Major Depressive Disorder (MDD), including the molecular basis of the remission phase, is crucial for understanding the neurobiology of this disease. A greater risk of a relapse of MDD during remission overlaps with higher sensitivity to stress, a major risk factor of this pathology [1], and is associated with higher susceptibility to MDD and resilience to this disease [2,3]. Currently, little is known about molecular changes during the remission phase and the role of inter-individual differences in the stress response [4,5,6].

Both genetic and epigenetic mechanisms have been shown to regulate individual vulnerability to stress-related MDD and are associated with a higher rate of relapse [7]. Recent findings suggest that molecular and physiological mechanisms associated with temporary remission from MDD syndrome can be distinct from those related to the manifestations of this disorder [8,9]. Unveiling these mechanisms can elucidate the biological basis of the vulnerability of patients to upcoming episodes of MDD relapse and thus develop potential therapeutic and preventive approaches.

Several molecular markers associated with the remission of depression have recently been identified. Patients with MDD in the remission phase revealed increased blood levels of inflammatory markers [10], decreased serotonin transporter binding in platelets and lymphocytes, hypercortisolemia, and hypocholesteremia, the reduced concentration of brain-derived neurotrophic factor (BDNF), and the decreased phosphorylation of cAMP response element-binding protein (CREB) [8,11]. Recent studies have reported links between markers of DNA damage [12], epigenetic markers of DNA methylation [9], and recovery from MDD. It has been suggested that remission in patients with MDD is associated with molecular processes regulating cell membrane properties, protein mobility, binding, and functions, for example, via changes in specific phosphatidylcholines and sphingomyelins [13,14], brain-specific gangliosides [15], and post-translational palmitoylation processes [16,17], which are critically involved in the regulation of multiple signaling processes and receptor functioning [18].

In particular, the post-translational modification of molecules via the palmitoylation of proteins via the thioester linkage of a 16-carbon fatty acid to a cysteine residue, an important regulatory mechanism of protein function [19], was recently proposed to contribute to the mechanisms of depression and inter-individual differences in susceptibility to MDD [20]. Altered palmitoylation is involved in neurological diseases, such as schizophrenia, intellectual disability, Huntington’s disease, cancer, and metabolic disorders [21,22,23,24,25,26]. Recent preclinical studies in mice and rats have revealed its role in the stress response [20]. The discovery of palmitoyl acetyltransferases containing a highly conserved core zinc finger DHHC domain (Asp-His-His-Cys) revealed their high substrate specificity for neuronal proteins, which are important for specific functions in CNS and neuropsychiatric conditions [27,28,29]. They play critical roles in synaptic plasticity and neuronal excitability [30,31,32]. DHHCs have been shown to change the mobility, binding, and functional properties of proteins and consequently regulate the trafficking and activity of signaling molecules, neurotransmitter receptors, synaptic scaffolding proteins, neurite outgrowth and guidance, neuronal differentiation, and synaptic vesicle release [30,33].

DHHCs moderate the palmitoylation of plasticity factors, whose roles in the mechanisms of stress and MDD are well known [34,35]. For example, DHHC8 and DHHC5 modify the postsynaptic targeting of postsynaptic density-95 (PSD-95) [33,36,37]. The knockdown of DHHC8 in cultured mouse cerebellar Purkinje neurons suppressed the palmitoylation of proteins that interact with C-kinase 1 (PICK1), leading to long-term synaptic depression [29,32]. DHHC9, which catalyzes protein S-acylation, was shown to be involved in normal brain development; a dysregulation of DHHC9 is associated with X-linked intellectual disability and increased epilepsy risk [38]. An association between the DHHC8 gene located in the 22q11 microdeletion region and schizophrenia has been reported [24,39,40,41,42]. DHHC8 polymorphisms are associated with smooth eye movements, a feature of schizophrenia [43], as well as cerebellar long-term depression [32].

Earlier studies showed that mice with a genetic deficit in DHHC8 display impaired pre-pulse inhibition, compromised contextual fear-related exploration, decreased sensitivity to an N-methyl-D-aspartate (NMDA) receptor blocker, lowered the density of dendritic spines, and diminished axonal branching [24]. These abnormalities have been implicated in the neurobiology of MD [44], which led to the hypothesis of the role of DHHC8 in this disorder. DHHC7 regulates the activities of NCAM140 and NCAM180 [45]. Thus, the important plasticity factors involved in the mechanisms of MDD are regulated by palmitoylation. A recent study showed the role of DHHC14 in catalyzing the palmitoylation of RAS, contributing to important cellular processes [46]. The palmitoylation of the 5-hydroxytryptamine-1A (5HT-1A) receptor, a key player in the serotonergic mechanisms of MDD, was shown to be altered by DHHC3 [33,47] and DHHC21 [1,20]. Recently, the downregulation of DHHC21 in the prefrontal cortex of mice that are predisposed to stress-induced depressive-like syndrome was shown to be associated with a reduced palmitoylation of serotonin receptor 5-HT1A, which is crucial for its signaling function. Reduced DHHC21 expression and 5-HT1A palmitoylation have also been observed in a mouse fear-conditioning paradigm [20].

Generally, excitatory and inhibitory synaptic functions that are dependent on the gamma-aminobutyric acid (GABA)-synthesizing enzyme GAD65 and synaptotagmin can be moderated by DHHCs [48]. Functional changes in plasticity factors and serotonergic and GABAergic mechanisms mediate the roles of DHHCs in neuropsychiatric disorders. Overall, genetic abnormalities of DHHC8, DHHC14, DHHC14L, DHHC17, DHHC9, DHHC12, and DHHC15 have been implicated in Huntington’s disease, Alzheimer’s disease, schizophrenia, and X-linked mental retardation [24,33,39,49,50].

The goal of this study was to investigate the potential molecular mechanisms underlying the phase of MDD remission and the role of DHHCs in these processes. Therefore, we used a mouse paradigm of stress-induced anhedonia [51] in which chronic stress exerts a hedonic deficit in the majority, but not all mice, in the sucrose test [51,52,53,54]. Stressed non-anhedonic (resilient) mice were used as internal controls for the effects of stress, which were not related to the depressive-like phenotype. In this model, the chronic administration of compounds with antidepressant properties decreased a proportion of anhedonic mice among stressed mice [54,55], which was also shown for classic antidepressants, as well as dicholine succinate (DS), an insulin receptor sensitizer that is used as adjunctive therapy in depressed patients [56,57,58]. Hence, we applied the chronic administration of imipramine (7 mg/kg/day), or DS (25 mg/kg/day), and stressed mice were studied for floating behavior in the forced swim test, exploratory rears in the novel cage test, and memory scores in contextual and cued fear conditioning as described elsewhere [52]. These behavioral experiments were performed during the 1st week following the termination of the stress procedure as an anhedonic state was found to persist during this post-stress time window [52,55]. The repeated sucrose test, as well as the Morris water maze test, were performed between the 3rd and 4th weeks post-stress, corresponding to the phase of remission. Thereafter, we used transcriptome analysis of the hippocampus, one of the key brain structures involved in emotional regulation during stress [59]. Transcriptome analysis of the hippocampi extracted during the remission period revealed inter-individual differences in gene expression with stress-induced susceptibility to hedonic deficit, as well as an altered hippocampal gene expression of six DHHCs. Subsequently, we studied the gene expression of six DHHCs that were significantly altered in the Illumina study: *Dhhc3*, *Dhhc7*, *Dhhc8*, *Dhhc13*, *Dhhc14*, and *Dhhc21* using RT-PCR. We found altered gene expression of *Dhhc8* and *Dhhc14* in the susceptible-to-MDD-like-syndrome subset of mice. 

## 2. Materials and Methods

### 2.1. Animals

Male C57BL/6J and CD1 mice (3.5 and 3 months old, respectively) were utilized in the study, with the former subjected to chronic stress and drug administration and the latter exclusively used for social defeat experiments. Male Wistar rats, aged 2–5 months, were used for predator stress procedure. All animals were obtained from Charles River (L’Arbresle, France) and housed according to previously described protocols [60]. C57BL/6J mice were individually housed for 10–14 days before the experiments; CD1 mice were housed in groups of five during the study, and rats were group-housed (five per cage) before being individually housed for the predation stress sessions. Animals were kept under an inverse 12 h light–dark cycle (lights on 20:00 h) so behavioral tests could be performed during the dark (active) phase of the physiological cycle of mice [61], and thus possible aberrations in behavioral analysis associated with unnatural lighting regimen could be precluded [62]. All experiments complied with the European Committees Council Directives, 2010/63/EU, ARRIVA guidelines, and received ethical approval of the Universidade Nova de Lisboa (http://www.nc3rs.org.uk/arrive-guidelines, accessed on 2 February 2024).

### 2.2. Study Flow

**Figure 1:**
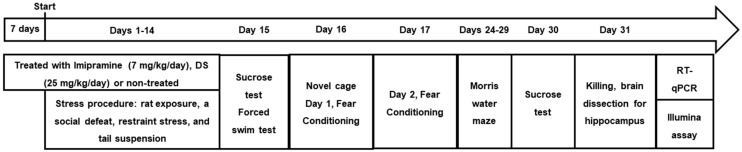
Experiment design. 3.5-month-old male C57BL/6J mouse was used for chronic stress study and drug administration. Drugs were administered starting seven days prior to stress and continued during a 14-day stress procedure, which comprised rat exposure, social defeat, restraint stress, and tail suspension. After stress induction, sucrose preference was assessed on day 15. On days 15–17, the forced swim, novel cage (day 16), and fear-conditioning tests (days 16 and 17) were performed. On days 24–29, mice were studied in the Morris water maze test using a two-trial protocol, and the sucrose test was performed on day 30; on day 31, all mice were sacrificed, and hippocampi were dissected and frozen. RNA was extracted and used for Illumina gene expression analysis and RT-qPCR (quantitative reverse transcription polymerase chain reaction).

Mice were studied for baseline sucrose test and weighed [51,52]; based on their values of body mass and sucrose preference, mice were assigned to a stress group (*n* = 58) and controls (*n* = 17) in a way that these initial parameters were balanced between the groups. In total, 75 animals were used (see Figure 1). Mice from the stress group were either not treated (S-NT, *n* = 20), or received imipramine via drinking water (7 mg/kg/day; S-imi, *n* = 19), or intraperitoneal injections of DS (25 mg/kg/day, S-DS, *n* = 19). Controls were untreated (C-NT, *n* = 7), or received imipramine (C-imi, *n* = 5) or DS (C-DS, *n* = 5). The sample size calculation was based on current DFG-compliant practice and performed as described elsewhere [52,55,57,60].

Drugs were administered as described elsewhere [57] starting 7 days prior to stress and continuing during a 14-day stress procedure, which comprised rat exposure, social defeat, restraint stress, and tail suspension [53,54,60] (also see below). After stress, on day 15, sucrose preference was evaluated [51,52]. Mice that displayed sucrose preference below 65% were considered anhedonic according to the validated criterion of anhedonia; the remaining mice were regarded as resilient to stress-induced anhedonia [52]. On days 15–17 (1st week following the stress procedure), forced swim, novel cage (day 16), and fear-conditioning tests (days 16 and 17) were performed [1,52,57,59,63]. After a one-week stress-free period, on days 24–29, the mice were studied in the Morris water maze model using a two-trial protocol, and the sucrose test was performed again on day 30. On day 31, all mice were sacrificed and the hippocampi were dissected and frozen [64]. RNA was extracted and used for the Illumina gene expression analysis (see below). RNA was partially converted to cDNA, RT-PCR was performed using primers for *Dhhc3*, *Dhhc7*, *Dhhc8*, *Dhhc13*, *Dhhc14*, and *Dhhc21*, and the housekeeping gene *Gapdh*, and gene expression was normalized to C-NT values [53,54]. The experimenters were blinded to the group assignments for all assays.

### 2.3. Stress Protocol

The 14-day stress procedure comprised of a nighttime rat exposure and the daytime use of three stressors: restraint stress, tail suspension, and social defeat, a combination of two of which was employed in a semi-random manner (for details, see below). Between 09:00 and 18:00, two stressors per day were employed in the following variances: social defeat for 30 min and restraint stress for 2 h, social defeat for 30 min and tail suspension for 6 min, or restraint stress for 2 h and tail suspension for 6 min. The minimal inter-session interval was at least 4 h.

#### 2.3.1. Rat Exposure While in a Small Container

Mice were placed inside transparent cylindrical glass containers (15 cm × Ø 8 cm) and introduced into the rat cage for exposure sessions lasting 15 h (conducted between 18:00 and 09:00). The containers, made from customized transparent plastic, featured perforated covers smaller than 0.5 cm in diameter, ensuring the mice were physically protected from the rats while maintaining visual and olfactory contact [52,65]. During weekends, the mice remained in their home cages, which were positioned above the rat cages.

#### 2.3.2. Tail Suspension Stress

Tail suspension stress was performed during the dark phase of the animals’ light cycle using a tail suspension system (Bioseb, Vitrolle, France). To prevent animals from observing or interacting with each other, each mouse was suspended within its own three-walled compartment, whose width and depth were sufficiently sized such that the mouse could not make contact with the walls. A suspension bar (100 cm, Ø 1 cm) was used to suspend the tail of each mouse [66]. Mice were suspended by their tails for approximately 6 min per session with adhesive tape 50 cm above the floor [57,63].

#### 2.3.3. Social Defeat Stress

Social defeat stress was induced during the dark phase under red light to provide visibility for monitoring interactions. Aggressive CD1 mice, pre-selected based on their ability to initiate attacks within 60 s without causing injury, were introduced to the home cages of stressed mice. These confrontations were set up in home cages to amplify the long-term effects of stress. A variation in the procedure involved learning the defeated mice in continuous contact with the olfactory cues of the aggressive intruder, creating a chronic psychological stressor with intermittent physical encounters. Each session lasted approximately 30 min, following established protocols [67,68]. During these sessions, stressed mice commonly exhibited behaviors, such as fleeing, adopting submissive postures, and vocalizing. Aggressive encounters were closely monitored to prevent physical harm, and the CD1 mice showing excessive aggression were immediately removed.

#### 2.3.4. Restraint Stress

The animals were placed inside a plastic tube with multiple air holes (internal diameter, 26 mm) for 2 h during the dark phase of the light cycle and kept in a dark experimental room [69]. Small pieces of facial tissue were inserted in the tubes to restrict animals’ activity [70].

### 2.4. Behavioral Tests

#### 2.4.1. Sucrose Test

Animals were given two bottles for 8 h, offering a free choice between a 1% sucrose or normal tap water. No prior deprivation of food or water was implemented before the test. To minimize the spillage of liquids during sucrose test, bottles were filled in advance (during preceding day or evening) and kept in the upside-down position for at least 12 h prior to testing. In order to balance the air temperature between the room and the drinking bottles, they were kept in the same room where the testing took place. This measure precludes the physical effect of liquid leakage resulting from increasing air temperature and pressure inside the bottles when they are filled with liquids, which are cooler than the room air. The bottles were weighed at the start and at the end of the experiment to calculate consumption. The test began at the onset of the dark (active) phase of the animals’ cycle. To prevent possible side preference in drinking behavior, the positions of the bottles were swapped after 4 h, midway through the test. The use of the applied variant of the sucrose test protocol allowed us to omit the training session; the error of the measurement of liquid intake was shown to not exceed 0.1 mL. Other testing conditions were consistent with previously described protocols [52,55,70]. The 1% sucrose solution was used both during baseline measurements and after the chronic stress exposure. Sucrose preference was calculated using the formula:

Sucrose Preference = [V (Sucrose solution)/V (Sucrose solution) + V (Water)] × 100%

A sucrose preference below 65% on the 14th day of continuous stress was considered a criterion of anhedonia. This threshold was established because none of the control animals showed a preference below 65% at that time point of the experiment. Mice with a sucrose preference below 65% were classified as anhedonic, while those with a preference above 65% were categorized as resilient to stress-induced anhedonia. Previous results confirmed that mice meeting this criterion exhibited depressive-like behaviors [52].

#### 2.4.2. Forced Swim Test

The forced swim test was performed as previously described [52]. A large square pool (21 cm × 42 cm × 15 cm) illuminated with red lighting was used, the water temperature was maintained at 30 °C, and the water height was 10 cm. This modified version has been shown to prevent behavioral artifacts caused by chronic stress-induced hyperlocomotion. A single swimming session lasted for 2 min. The absence of any directed movement of the heads and bodies of the animals for more than 3 s was determined to be floating. The latency of the first episode and the duration of floating were scored. Visual scoring was validated as described previously [52] using the Ethovision Program 6.95 (Noldus, Wageningen, The Netherlands).

#### 2.4.3. Novel Cage Test

The mice were placed in a clear plastic cage (14 × 21 × 27 cm) with a small amount of fresh litter. The number of rears was measured for 5 min under red light, as described elsewhere [52].

#### 2.4.4. Fear Conditioning

All groups of mice were analyzed using a contextual fear-conditioning learning test, a well-established paradigm for hippocampus-dependent memory in rodents, and auditory conditioning, which is predominantly an amygdale-dependent form of learning [59,63]. Three days after the stress procedure, mice were subjected to a training session. On the fourth day, they were tested for contextual and cued memory recall with an inter-test interval of 6 h (24 and 30 h after training, respectively). The procedure was performed as previously described [63]. The apparatus consisted of a transparent plastic cubicle (25 cm × 25 cm × 50 cm) with a stainless-steel grid floor (33 rods, 2 mm in diameter; Technosmart, Rome, Italy). An alternating single electric current (AC, 50 Hz, Evolocus, Terrytown, NY, USA) was delivered after a 2 min acclimatization of a mouse to a chamber and was co-terminated with a 2 s tone produced by a sound generator (80 dB, 3000 Hz). Immediately after the delivery of the current and tone, the mice were placed back in the home cage. For the contextual memory recall session, the animals were placed in the conditioning apparatus used in the training session 24 h later. Freezing behavior is defined as a complete lack of movement in addition to respiration. It was scored by visual observation during two tests of memory recall; its occurrence was assessed every 10 s for 180 s; each 10 s score was assigned to a freezing or non-freezing period; and the percentage of time spent freezing was calculated. For the auditory memory recall session, three hours later, mice were placed into a new context (a cage 23 × 18 × 35 cm situated in a different room) for 360 s: the first 180 s without and the second 180 s with the tone presentation. Freezing behavior was scored as described above.

#### 2.4.5. Morris Water Maze

The inner walls of the tank (Ø = 100 cm, 70 cm high) were covered with A4-sized visual cues of different shapes at positions such as northeast (NE), southeast (SE), southwest (SW), and northwest (NW). On four consecutive days, the mice were placed for 120 s in a pool of water mixed with milk twice, with an interval of 90 min between them. A platform (5 × 5 cm) was submerged 1 cm below water, at the center of the sector, and divided into four positions of the platform that could be placed at NE, SW, and NW of SE, to prevent possible confounds [71,72]. The maximum number of mice examined from each group was eight. The four previously mentioned starting points were randomly assigned to the animals as starting locations. Once a mouse either reached the platform or was placed on it after the trial time had expired, it remained on the platform for 30 s. Escape latency was recorded for each trial. On day 5, a probe test was performed, where mice were allowed to swim in the pool, and the time they spent in the ‘correct’ quadrant at which the platform was located during the training period was registered. The percentage of time spent in the ‘correct’ quadrant relative to the total test duration was calculated as an indicator of spatial memory. All trials were recorded and scored offline using Ethovision Program 6.95 (Noldus, Wageningen, The Netherlands). In all trials, the animals were brought to the pool room for a 40 min acclimatization. Two setups were simultaneously used in the adjustment rooms.

### 2.5. Administration of Compounds

Imipramine (Sigma-Aldrich, St. Louis, MO, USA) was dissolved in tap water, with the solution freshly prepared every 2–3 days. Since imipramine is sensitive to light, bottles containing the solution were covered with aluminum to provide protection. The concentration of imipramine in the drinking water was calculated based on the previously determined average daily water intake in C57BL/6J mice, which was approximately 3 mL, on an average mice weight of 28 g, and on the dosage of treatment 7 mg/kg/day [72]; final concentration of imipramine in the solution was about 63 mg per 1 L of tap water. The dosage of imipramine was set at 7 mg/kg/day as previous studies have shown that chronic administration of this antidepressant at higher doses compromises behavioral and physiological measures in naïve mice [73,74]. Imipramine administration began one week before the onset of the stress procedure and continued throughout the chronic stress period.

DS (C_14_H_32_N_2_O_6_), obtained from Buddha Biopharma Oy Ltd. (Helsinki, Finland), was dissolved in water for injection and administrated via daily intraperitoneal (i.p.) injections at a dose of 25 mg/kg/day for seven consecutive days. This treatment regimen has been shown to produce memory-enhancing effects and neurochemical effects lasting for at least two weeks in mice and rats [75]. Injection volumes for DS and vehicle were calculated as 0.01 mL per g of body weight.

### 2.6. Culling of Mice and Tissue Collection

Mice were euthanized by the terminal anesthesia using CO_2_ and isoflurane, following previously established protocol [60]. After perfusion with ice-cold NaCl, the brain was extracted, and the hippocampi were microdissected on dry ice, immediately frozen, and stored at −80 °C until further use.

### 2.7. Illumina Gene Expression Profiling

Total mRNA was isolated from all experimental samples using RNeasy Lipid Tissue Mini Kit (Qiagen, Hilden, Germany). Gene expression profiling of the hippocampus from all experimental groups was conducted using Illumina technology (IntegraGen, Evry, France), as described previously [75,76]. Samples were randomly assigned to chips, with the condition that no two samples from the same group were placed on the same chip, minimizing bias between the experimental groups. Standard analysis protocols were used to assess the microarray data, beginning with an evaluation of the overall data quality and the statistical evaluation of differentially expressed genes. After confirming sufficient data quality, the Gene Chip Operating System was employed to compute the signal intensities, detection calls, and associated *p*-values for each transcription array. Gene expression levels were normalized to the housekeeping gene, glyceraldehyde-3-phosphate dehydrogenase (Gapdh), which demonstrated stable expression [71], and calculated as fold changes relative to the control drug-naïve non-stressed group. Illumina data were imported into the Partek Genomics Suite and quantile-normalized. Arrays identified as outliers through PCA were excluded from the dataset. Group comparisons were conducted in the Partek Genomics Suite using ANOVA with appropriate contrasts, and *p*-values were adjusted for multiple testing using the step-up False Discovery Rate (FDR). The following criteria were used to select differentially expressed genes: strict: FDR < 0.05 and |fold change| > 2; medium: FDR < 0.1 and |fold change| > 1.5; loose: unadjusted *p*-value < 0.001 and |fold change| > 1.3; and very loose: unadjusted *p*-values < 0.01 and no fold change threshold (only used when more stringent selection criteria yielded zero or very few hits). In this study, the ‘medium’ criteria were applied. Each experimental group consisted of 5 animals.

### 2.8. Quantitative Real-Time PCR

RNA extraction was performed from microdissected snap-frozen hippocampi using RNeasy RNA extraction kit with DNaseI treatment (Qiagen, Hilden, Germany), as previously described [54]. Isolated mRNA was used for cDNA synthesis; 1 μg total RNA was converted into cDNA using QuantiTect Reverse Transcription Kit (Qiagen, Hilden, Germany). qRT-PCR was carried out using the SYBR Green master mix (Bio-Rad Laboratories, Philadelphia, PA, USA) and the ProFlex PCR system (Thermo Fisher Scientific, MA, USA). qRT-PCR was performed in a 10 μL reaction volume containing a SYBR Green master mix (5 μL), RNase-free water (3 μL), specific forward and reverse primers used at the concentration 20 pmol/μL (1 μL), and cDNA (1 μL). Gapdh was selected as a reference gene since in previous experiments it was observed relatively low variability in its brain expression in similar studies [54,57]. The initial denaturation step for qRT-PCR was at 95 °C for 4 min followed by 40 cycles of denaturation at 95 °C for 20 s, and annealing was at 54 °C for 90 s. The PCR efficiency was assessed using Linreg, and melting point for each PCR was run for each PCR. Sequences of all primers used are listed in Table S1 (see Supplementary Materials). All samples were run in triplicate. Results of the real-time PCR measurements are represented as Ct values, where Ct is defined as the threshold cycle of PCR at which amplified product was 0.05% of normalized maximal signal. Data were normalized to Gapdh mRNA expression and calculated as relative-fold changes compared to control vehicle-treated mice, as described elsewhere [54,57,77].

### 2.9. Statistics

GraphPad Prism (v.5; San Diego, CA, USA) was used for data analysis by incorporating one- and two-way ANOVA and Bonferroni or Tukey’s post-hoc tests, where appropriate. Qualitative data were analyzed using Fisher’s exact test. The confidence level was set at 95% (*p* < 0.05). Data are shown as the mean ± SEM.

## 3. Results

### 3.1. Stressed Mice Split into Anhedonic and Resilient Subsets and Display Distinct Behavioral Changes

**Figure 2:**
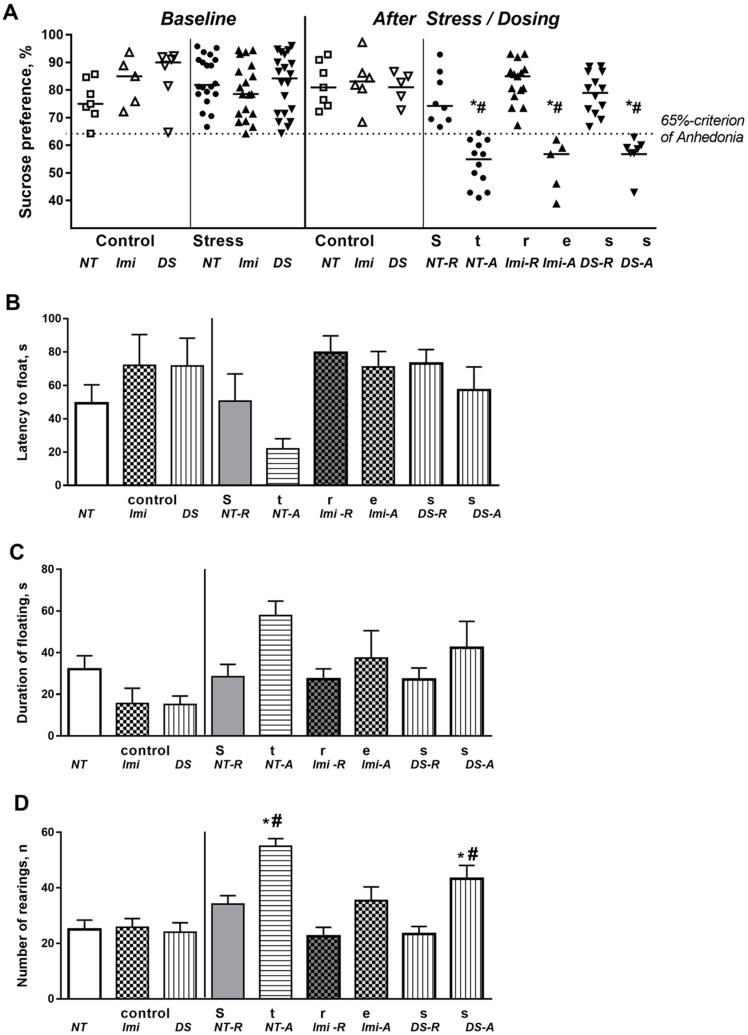
Measured parameters of hedonic state and emotionality: (**A**) sucrose preference, (**B**) latency to float, (**C**) duration of floating, and (**D**) number of rears (see the text). * vs. respected controls, # vs. respected resilient groups. Symbols in subfigure (**A**)—open symbols: non-stressed (control) roups; closed symbols: stress groups; circles: non-treated groups; triangles directed up: Imi-treated groups; triangles directed down: DS-treated groups. NT: non-treated group; NT-R: non-treated resilient group; NT-A: non-treated anhedonic group; Imi: imipramine-treated group; Imi-R: imipramine-treated resilient group; Imi-A: imipramine-treated anhedonic group; DS: DS-treated group; DS-R: DS-treated resilient group; DS-A: DS-treated anhedonic group. (C-NT group, *n* = 7, C-Imi group, *n* = 5, C-DS group, *n* = 5, S-NT-R group, *n* = 8, S-NT-A group, *n* = 12, S-Imi-R group, *n* = 14, S-Imi-A group, *n* = 5, S-DS-R group, *n* = 12, S-DS-A group, *n* = 7.) Bars are mean ± SEM.

There were no significant group differences in sucrose preference between mice before stress (F = 0.91, *p* = 0.48, one-way ANOVA; Figure 2A). After stress, a significant effect (F = 12.39, *p* = 0.0011, two-way ANOVA) was observed, but no treatment effect or stress × treatment interaction was found (F = 2.74, *p* = 0.28, and F = 2.18, *p* = 0.37, respectively; Figure 2A). In accordance with the applied anhedonia criterion, the stressed groups were stratified into two subgroups. In the S-NT group, 60.0% of mice displayed ‘anhedonia’ (A), while among S-Imi and S-DS mice, this measure constituted 26.31% and 35%, respectively. The remaining mice were considered ‘resilient’ (R). The non-treated, imipramine-treated, and DS-treated anhedonic groups all had significantly lower sucrose preference than the non-treated control group (all *p* < 0.0001) and respective resilient groups (all *p* < 0.0001). A subsequent analysis focused on the comparison of these subgroups between each other and against the NT-C group.

In the forced swim test, significant group differences were found in the latency to float (F = 3.095, *p* = 0.005, one-way ANOVA; Figure 2B). The duration of floating differed significantly between groups (F = 3.31, *p* = 0.03, Figure 2C). No other differences were observed in the post hoc tests for both parameters (*p* > 0.05). The number of rears in the novel cage test was significantly different between groups (F = 13.53, *p* < 0.0001, one-way ANOVA, Figure 2D). This score was significantly higher in the NT-A and DS-A groups, but not in the Imi-A group, than in the C-NT group (*p* < 0.0001, *p* = 0.0155, and *p* = 0.619, respectively; Tukey’s test), as well as compared to the respective resilient groups (*p* = 0.0013, *p* = 0.0019, and *p* = 0.296, respectively).

**Figure 3:**
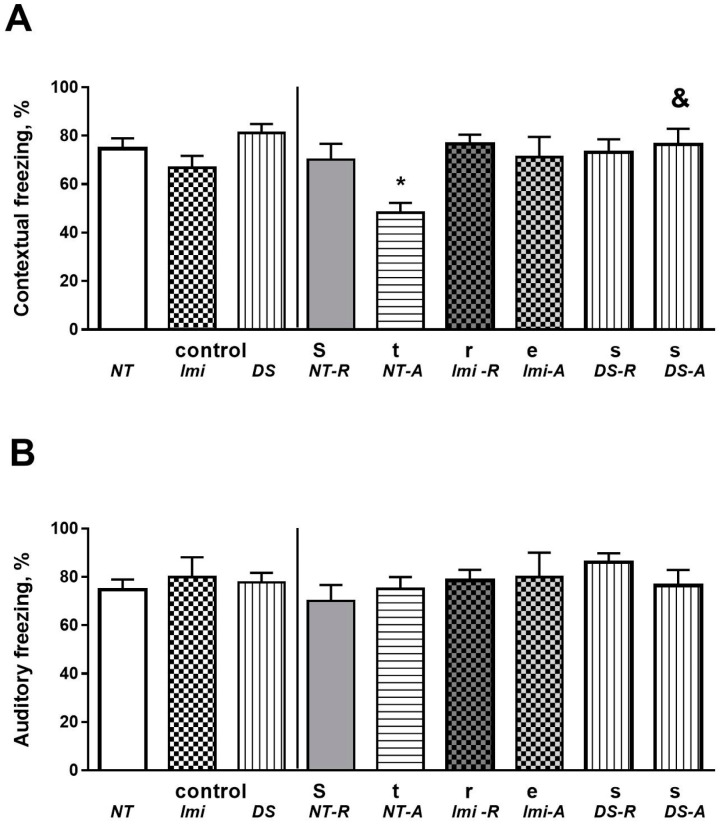
Scores of freezing behaviors in recall sessions for (**A**) contextual and (**B**) auditory learning (see the text). *p* < 0.05, * vs. respected control group, & vs. S-NT-A group. (C-NT group, *n* = 7, C-Imi group, *n* = 5, C-DS group, *n* = 5, S-NT-R group, *n* = 8, S-NT-A group, *n* = 12, S-Imi-R group, *n* = 14, S-Imi-A group, *n* = 5, S-DS-R group, *n* = 12, S-DS-A group, *n* = 7). For group labels, see Figure 2. Bars are mean ± SEM.

In the fear-conditioning model, freezing scores of contextual memory were significantly different between groups (F = 4.06, *p* = 0.0006, one-way ANOVA; Figure 3A). S-NT-A animals showed a significant reduction in this measure compared to C-NT mice (*p* = 0.0145, Tukey’s test), and there was a strong trend for a decrease in this measure compared to S-NT-R animals (*p* = 0.0584). S-imi-A and S-DS-A mice did not show such changes (*p* > 0.05). At the same time, S-DS-A, but not S-Imi-A, mice showed significantly higher freezing scores than the S-NT-A group (*p* = 0.068 and *p* = 0.125, respectively).

The freezing scores of auditory fear memory did not differ significantly between the groups (F = 0.74, *p* = 0.659, one-way ANOVA; Figure 3B).

### 3.2. Stressed Mice Spontaneously Recover from Hedonic Deficit and Reveal Unchanged Spatial Memory

**Figure 4:**
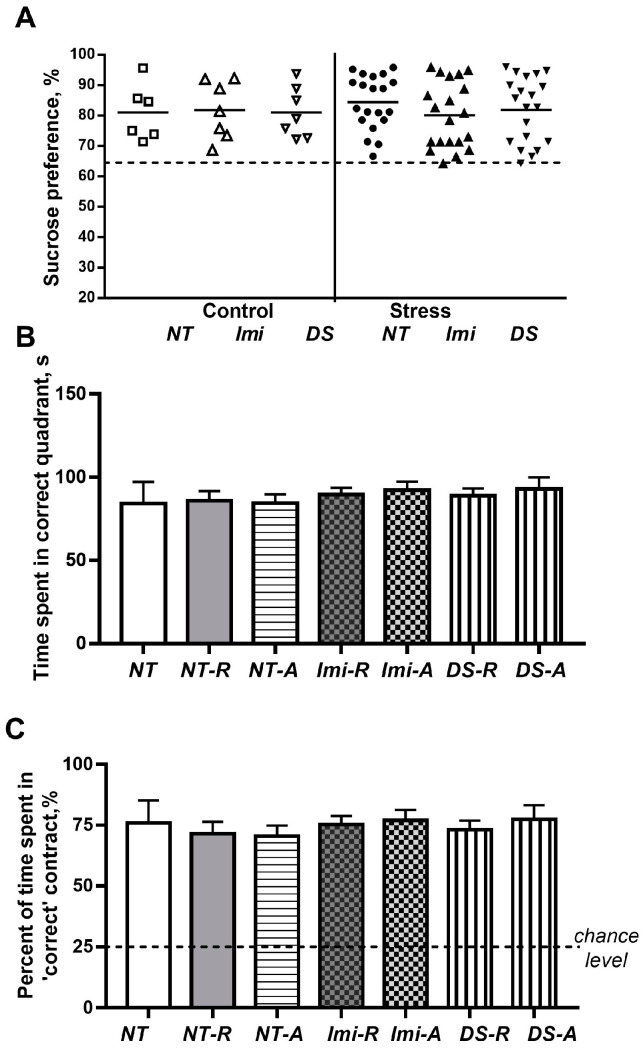
Sucrose test data and a recall of spatial memory in the Morris water maze at ‘recovery’ post-stress phase. No significant group differences were found in (**A**) the sucrose preference, as well as in (**B**) time spent in the ‘correct’ quadrant, and (**C**) preference for this quadrant in the Morris water maze. For group labels, see Figure 2. (Symbols in subfigure (**A**)—open symbols: non-stressed (control) roups; closed symbols: stress groups; circles: non-treated groups; triangles directed up: Imi-treated groups; triangles directed down: DS-treated groups. C-NT group, *n* = 7, C-Imi group, *n* = 5, C-DS group, *n* = 5, S-NT-R group, *n* = 8, S-NT-A group, *n* = 12, S-Imi-R group, *n* = 14, S-Imi-A group, *n* = 5, S-DS-R group, *n* = 12, S-DS-A group, *n* = 7; see also the text, Section 2.4.5.) Bars are mean ± SEM.

At the ‘recovery phase’, we found no significant group differences in the sucrose preference (F = 0.407, *p* = 0.824, one-way ANOVA, Figure 4A) suggesting that susceptible mice have recovered from a compromised deficit in hedonic deficit spontaneously. During this post-stress period of the experiment, no significant group differences were found in the latency to escape in the Morris water maze on days 1–4 (all *p* > 0.05;. During the probe trial, all groups demonstrated similar time spent in the ‘correct’ quadrant and preference for this quadrant as no significant group differences were shown in these measures (F = 0.32, *p* = 0.924 and F = 0.285, *p* = 0.942, respectively, one-way ANOVA, Figure 4B,C). For all groups, the preference for the ‘correct’ quadrant was significantly higher than a 25%-chance level (*p* > 0.05, for all groups).

These data suggest a lack of deficiency in spatial hippocampus-dependent memory in susceptible mice that recovered their sensitivity to reward in the sucrose test. These results also rule out the possibility that other general factors, such as pharmacological treatment, had an impact on the acquisition of escape responses and spatial learning in the Morris water maze paradigm.

### 3.3. Transcriptome Analysis of DHHC Expression in the Hippocampus

The results of transcriptome analysis of DHHC expression in the hippocampus are presented in Table 1. We found that the expression of several DHHCs was significantly altered in comparison to the untreated control group in both pharmacologically naïve and dosed animals that were categorized into resilient or anhedonic subgroups (Table 1). Interestingly, in only one case, we found a significant decrease in expression *(Dhhc4*), and most of the changes in expression were found in resilient groups that demonstrated elevated expression of several DHHCs. For further RT-PCR experiments, we selected six DHHCs whose expression was significantly altered in at least three out of six experimental groups: *Dhhc3*, *Dhhc7*, *Dhhc8*, *Dhhc13*, *Dhhc14*, and *Dhhc21*. Notably, the latter molecule was previously reported to play a causal role in the mechanisms of stress resilience as it was found to be downregulated in anhedonic mice studied in a similar stress model [20]. Here, we found increases in its expression in both anhedonic and non-treated and DS-treated resilient groups of mice, where the latter groups showed pronounced changes (Table 1); in particular, an increase in Dhhc21 expression in the anhedonic subgroup was the largest among the DHHCs. The selected DHHCs were examined for gene expression using RT-PCR.

### 3.4. Other Changes in the Illumina Assay

Illumina assay revealed a significant decrease in genes encoding serotonin transporter *(Sert*), serotonin receptor *5-ht2a*, tryptophan hydroxylase 2 (*Tph2*), DOPA decarboxylase (*Ddc*), and brain-derived neurotrophic factor (*Bdnf*), as well as an increase in glycogen synthase kinase 3β (*Gsk-3β*) in stressed groups. Specifically, the expression of brain-derived neurotrophic factor (*Bdnf*) and its receptor *Ntrk2* was significantly higher in the non-treated resilient group than in an all of the other anhedonic groups regardless of treatment (for *Bdnf*, mean value 1.21 vs. 1.06 in the non-treated group, 0.96 in the DS-treated group, and 1.11 in the imipramine-treated group, all *p* < 0.05; for *Ntrk2*, 1.34 vs. 0.97 in the non-treated group, 0.98 in the DS-treated group, and 0.94 in the imipramine-treated group, all *p* < 0.05). Next, glycogen synthase kinase 3β (*Gsk-3β*) expression was shown to be increased in non-treated mice, both resilient and anhedonic, but lower in groups treated with imipramine or DS (mean value 1.49 and 1.41 in the non-treated stress-resilient and anhedonic groups, respectively, vs. 1.16 in the imipramine-treated group and 0.94 in the DS-treated group, all *p* < 0.05). The expression of the gene-encoding serotonin reuptake transporter (*Sert*) was lower in the non-treated stress groups than in the imipramine- or DS-treated groups (mean value 0.79 and 0.82; non-treated stress-resilient and anhedonic groups, respectively, vs. 1.16 in the imipramine-treated group and 1.04 in the DS-treated group, all *p* < 0.05).

Non-treated stress-resilient mice and stressed DS-treated mice showed an increased expression of *5-ht2a*, which was not observed in imipramine-treated or non-treated anhedonic mice (mean value 0.96 in anhedonic non-treated mice and 1.02 in imipramine-treated mice vs. 1.21 in non-treated stress-resilient mice, *p* < 0.05, and vs. 1.15 in DS-treated mice, *p* > 0.05). The expression of tryptophan hydroxylase 2 (*Tph2*) was significantly decreased in both anhedonic and resilient non-treated groups, which was not observed in mice treated with imipramine or DS (mean value 0.51 and 0.50 in non-treated stress-resilient and anhedonic groups, respectively, vs. 1.12 in the imipramine-treated group and 1.05 in the DS-treated group, all *p* < 0.05). The same trends were observed for *Ddc* gene (mean value 1.12 in imipramine-treated mice and 1.19 in DS-treated mice vs. 0.78 in non-treated anhedonic mice, *p* < 0.05, and vs. 0.90 in non-treated stress-resilient mice, *p* > 0.05). Finally, imipramine-treated mice demonstrated increased insulin receptor expression that was not observed in both dicholine succinate (DS)-treated and non-treated groups (mean value 1.26 in the imipramine-treated group vs. 0.96 in the DS-treated group and 1.04 and 1.08 in the non-treated stress-resilient and anhedonic groups, respectively, all *p* < 0.05).

### 3.5. Hippocampal Gene Expression of Selected DHHCs

**Figure 5:**
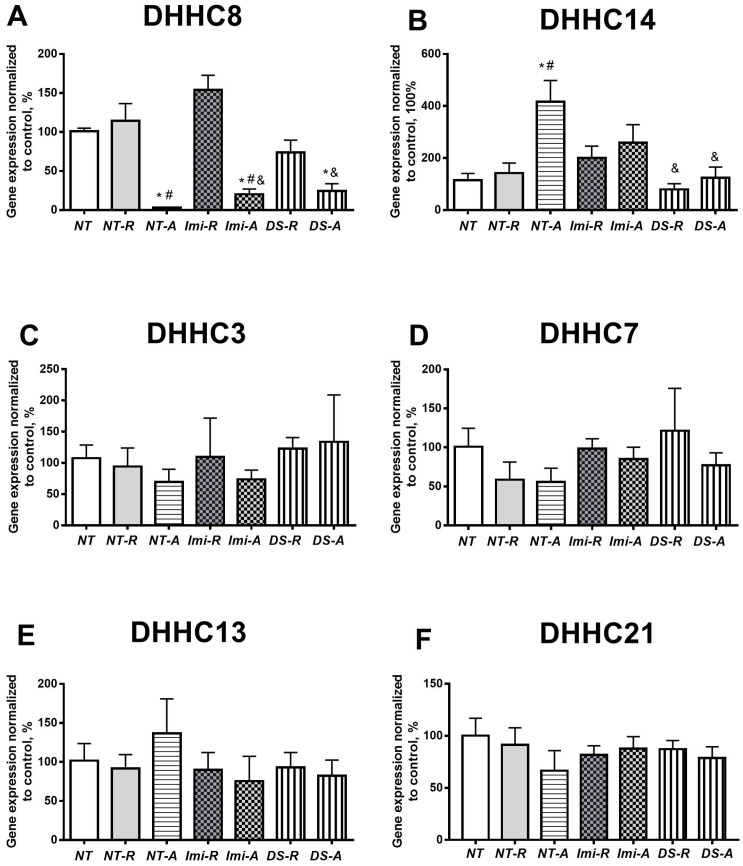
Hippocampal expression of six DHHCs. There was an overall significant difference between the groups in gene expression for (**A**) *Dhhc8* and (**B**) *Dhhc14* (see the text). No group differences were found for (**C**) *Dhhc3*, (**D**) *Dhhc*7, (**E**) *Dhhc13*, or (**F**) *Dhhc21*. *p* < 0.05, * vs. C-NT group, *p* < 0.05, # vs. S-NT-R groups, and *p* < 0.05, & vs. S-NT-A group. For group labels, see Figure 2. Bars are mean ± SEM.

*Dhhc8* mRNA levels were significantly altered (F = 6.23, *p* = 0.002, one-way ANOVA); this measure was significantly decreased in all anhedonic groups, as compared to C-NT and S-NT-R groups (*p* < 0.05, Tukey test, Figure 5A). S-NT-A mice showed lower Dhhc8mRNA levels than S-imi-A and S-DS-A mice (*p* < 0.05). The expression of *Dhhc14* was significantly different between the groups (F = 5.284, *p* = 0.0009, one-way ANOVA), and the anhedonic non-treated group displayed a significant increase in the gene expression of *Dhhc14* (*p* < 0.01, Figure 5B). In this group, the *Dhhc14* mRNA levels were significantly higher than those in the S-DS-R and S-DS-A groups (*p* = 0.0010 and *p* = 0.0054, respectively; Figure 5B). We found no group differences in *Dhhc3*, *Dhhc13*, *Dhhc7*, and *Dhhc21* gene expression (*p* > 0.05, Figure 5C–F).

Because we have revealed a lack of effects of imipramine or DS, under the dosing conditions employed here, on behavioral and molecular parameters, including the lack of difference in gene expression of *Dhhc8* and *Dhhc14* (see Supplementary Figure S1), non-stressed control mice, Imi-C, and DS-C were omitted from further PCR analysis.

## 4. Discussion

The present study demonstrated a relationship between altered hippocampal *Dhhc8* and *Dhhc14* expression and the remission phase in mice vulnerable to MDD-like syndrome. Both drug-naïve anhedonic mice and animals that received antidepressant therapies of different classes, but that remained refractory to these treatments, showed a significantly lower hippocampal gene expression of *Dhhc8.* In contrast, the expression of *Dhhc14* in anhedonic mice was increased in comparison to controls; this increase was observed only in the drug-naïve anhedonic group. Gene expressions of *Dhhc3*, *Dhhc13*, *Dhhc7*, and *Dhhc21*, which were highlighted by the analysis of the Illumina transcriptome study, were not significantly changed. Resilient groups of mice did not show any changes in the hippocampal expression of DHHCs compared to non-stressed controls.

The induction of a depressive-like state in the employed model was defined by the occurrence of a drop in sucrose preference in a subset of mice below the lowest levels of this measure displayed by non-stressed control mice (65%). Previous behavioral, molecular, transcriptome, and metabolome studies have established the validity of this approach in the stratification of animals into MDD-susceptible (anhedonic) and resilient (non-anhedonic) subsets of mice [51,52,53]. Here, stressed mice showed trends of increased floating behavior, a sign of helplessness, and the lack of significant changes in this behavior was consistent with our previous observations [55,57]. This can be explained by the partial amelioration of depressive-like syndrome in the imipramine- and DS-treated subgroups of mice [52,57]. We also found hyperactive rearing behavior in the novel cage test, a sign of increased stress response [60], in all anhedonic groups. Again, this suggests that some behavioral aberrations were improved by antidepressant treatment, whereas others were not.

Anhedonic drug-naïve mice demonstrated a disrupted hippocampus-dependent memory in the contextual fear paradigm, whereas no memory deficits were found in pharmacologically treated anhedonic animals. In the cued fear-conditioning test, there were no signs of learning deficits in any experimental group. This suggests well-established specificity in the deficiency of learning and memory processes associated with MDD-like syndrome concerning the impairment of hippocampus-dependent functions [59].

At the recovery phase from MDD-like syndrome, we found no significant group differences in sucrose intake. Moreover, not a single animal, also from the anhedonic groups, showed a sucrose preference that was below the control values. These results are in line with our previous observations showing that the stress-induced anhedonic state in mice employed in this study can be observed for no longer than two weeks [55,57,75]. The lack of changes in memory acquisition in the Morris water maze further supports the observation of behavioral and functional recovery from an MDD-like syndrome in anhedonic groups of stressed mice. However, the persistence of lasting molecular changes, such as the downregulation of *Dhhc8* and the upregulation of *Dhhc14* gene expression in the hippocampus of anhedonic but not resilient mice, suggests that the former group might remain vulnerable to challenges that can re-instate the MDD-like syndrome. Indeed, earlier studies have shown that additional stress, such as restraint or rat exposure stress, can induce a state of anhedonia in animals that were previously defined as anhedonic [51,52,55]. Thus, selective changes in the hippocampal expression of *Dhhc8* and *Dhhc14* can underpin this susceptibility under MDD conditions during the remission phase.

To date, accumulated evidence suggests a role for DHHC8 in morphological plasticity, a mechanism of adaptive stress response, and learning [44]. As discussed above, DHHC8 was found to be a crucial regulatory factor of PSD-95, a major scaffolding protein that affects synaptic plasticity via BDNF/TrkB-mediated signaling, which is considered an important molecular mechanism of MD [76]. Additionally, DHHC8 regulates AMPA- and NMDA-glutamate receptor function [28,29,30,39,78]. Both receptors are critically involved in the mechanism of MDD [44]. On the other hand, the deletion of DHHC8 results in the decreased binding of the NR2B receptor subunit of the NMDA receptor [28,29,30], which plays a key role in the mechanisms of stress response and MDD [79]. As such, the compromised gene expression of *Dhhc8* in anhedonic mice may underlie the stress responsiveness associated with MDD-like syndrome. Ameliorated memory scores in the fear-conditioning paradigm, a form of hippocampus-dependent learning, were accompanied by the normalized gene expression of *Dhhc8* in this brain structure of anhedonic groups from DS- and imipramine-treated cohorts of stressed mice.

These data are in line with previous demonstrations of the key role of Dhhc8 in long-term synaptic depression [29,37], which can be underlined by PICK1 function and consequent aberrations in NCAM140 and NCAM180 expression [36], which are major players in synaptic plasticity. It is possible that a decrease in *Dhhc8* expression in anhedonic mice does not play a causal role in MD-like mechanisms but results from other neurochemical changes, such as the over-production of neuronal nitric oxide (nNO), a factor of stress and MDD [21], and NMDA-receptor-mediated excitotoxicity. Both mechanisms have been shown to suppress the palmitoylation of several important proteins, including PSD-95 [28,29,30].

As for DHHC14, the recent study showed the role of this DHHC in the catalyzing of the palmitoylation of RAS, contributing to important cellular processes [46]. DHHC14 is highly expressed in the hippocampus, regulating Kv1 potassium channels, thus exerting neurodevelopmental effects. The loss of DHHC14 decreases outward currents and increases action potential firing in hippocampal neurons [80]. A recent whole-genome sequencing study showed the crucial role of DHHC14 in antiviral resistance mechanisms [81]. Previous studies suggest that DHHC14 is involved in Huntington’s disease and some neurodegenerative conditions [33,40], as well as an increased risk of diabetes mellitus [82] and coronary artery disease [83], both comorbid with MDD disorder.

At the same time, we found no significant changes in the expression of other DHHCs examined by RT-PCR in the hippocampus of stressed mice. However, their potential role in stress- and depression-related behaviors has been suggested in several studies. For instance, DHHC13 deficiency in mice leads to anxiety, motor impairments, and altered brain bioenergetics through the reduced S-palmitoylation of Drp1 [84]. A deficit in DHHC7 was found to alter stress responses via mechanisms of synaptic plasticity and the brain microstructure in a sex-dependent manner [85]. The genetic deletion of DHHC3 has been shown to enhance the mechanisms of oxidative stress [86], an important pathogenic element of individual predisposition to stress-induced MDD syndrome [74,87]. The lack of changes in the hippocampal gene expression of these molecules in our study might be due to the structure-specific roles of these molecules in CNS functions, distinct from other DHHC dynamics of after-stress recovery in expression and other factors.

Similarly, the gene expression of *Dhhc21* in the hippocampus of anhedonic mice during the post-stress period of recovery from anhedonic syndrome was unaltered in our study. This suggests that this molecule is not involved in the molecular changes underlying remission from MDD-like syndrome. However, previously reported findings suggesting the role of DHHC21 in the mechanisms of depressive-like syndrome and anhedonia were observed in the prefrontal cortex [20]. The role of this brain structure during stress is distinct from that in the hippocampus. On the other hand, the lack of changes in the hippocampal expression of *Dhhc21* in the present study can further support the link between the anhedonic state and downregulation of DHHC21 since, in the current study, its expression was examined when no anhedonia was displayed by stressed mice.

Changes in the expression of genes encoding SERT, 5-HT2A, Tph2, DOPA, DDC, BDNF, and GSK-3β in the stressed groups, as shown in the Illumina assay, are in line with previous studies suggesting the role of these molecules in the mechanisms of MDD [77,88,89,90]. Importantly, these molecules have also been shown to be associated with the remission of MDD [91,92,93,94,95].

As it is discussed above, palmitoylation is a common reversible post-translational modification that is critically involved in the regulation of the functions of these molecules [18]. In this context, among the additional gene expression changes found, serotonin-related mechanisms have been studied the most [18]. Specifically, the palmitoylation of 5-HT1B [96], 5-HT4 [97], and 5-HT7 [98] may be associated with the development of anxiety and depression due to their roles in physiological and pathophysiological responses, and the palmitoylation of 5-HT1A receptors may be of the most importance since it is irreversible and insensitive to agonist stimulation [99]. The dopamine receptors D1, D2, D3, and D4 have also been shown to be palmitoylated [100,101,102,103], which may contribute to the development of depression and the remission phase of MDD since dopamine plays a crucial role in the regulation of executive function, reward, and motivation. The palmitoylation of AMPA [104] and NMDA receptors [105] may also play a crucial role in pathological behaviors, including depression, owing to their role in synaptic plasticity, synaptogenesis, and excitotoxicity. GABA type A receptors are also involved in various kinds of behavioral regulation and are the major inhibitory neurotransmitter receptors in the mammalian brain. Its γ2 subunit undergoes palmitoylation, which may significantly affect the functional regulation of the receptor [106].

Together, present and previous studies highlight the importance of protein palmitoylation in maintaining normal brain function and resilience to MDD syndrome suggesting that disruptions in DHHC brain expression activity may contribute to stress-related disorders and depression. Our findings demonstrate that DHHC-type protein acyltransferases may have broader implications in cellular stress responses and neuroplasticity, potentially connecting to stress and depression mechanisms. As such, targeting DHHCs or their products and substrates could potentially offer new therapeutic strategies for these conditions.

## 5. Conclusions

Although further studies are warranted to address the role of palmitoylation in MDD development and the mechanisms associated with the phase of remission of this disorder, the present data suggest the potential of agents that can alter palmitoylation and thereby generate a sustainable antidepressant-like effect. Since palmitoylation reversibly modulates important signaling factors, it can be an attractive target for MDD pharmacotherapy [23,31]. Such possibilities can be discussed in the context of other comorbidities of depression, such as diseases of the nervous system and metabolic disorders, given the broad regulatory spectrum of DHHCs [21,26]. However, the use of a single brain region and the lack of dynamic trajectories may be considered as limitations of this study. Yet, current and former studies suggest an important role for DHHCs in the mechanisms of individual susceptibility to stress and MDD syndrome.

## Figures and Tables

**Table 1:** Transcriptome analysis of DHHC expression in the hippocampus. The expression of *Dhhc3*, *Dhhc4*, *Dhhc5*, *Dhhc7*, *Dhhc8*, *Dhhc13*, *Dhhc14*, *Dhhc16*, *Dhhc17*, *Dhhc21*, and *Dhhc2*3 was significantly altered in the anhedonic and resilient, pharmacologically naïve, and dosed groups in comparison to respective control groups (labeled with apteryx). In all groups, *n* = 5. For *Dhhc3*, *Dhhc7*, *Dhhc8*, *Dhhc13*, *Dhhc14*, and *Dhhc21*, the expression was significantly altered in at least three groups (labeled in bold and underscored). These DHHCs were then examined for gene expression using RT-PCR. One-way ANOVA, * *p* < 0.05.

	S-NT-A	S-DS-A	S-Imi-A	S-NT-R	S-DS-R	S-Imi-A
**DHHC1**	1.042469947	1.097460004	1.182845106	1.088137954	1.025735695	1.006418503
**DHHC2**	1.132028935	1.098419853	1.175033359	1.140535814	0.848323881	0.969484222
**DHHC3**	1.130328882	1.049058653	1.116955408	1.59297701 *	1.410208834 *	1.213223399 *
**DHHC4**	0.798861998 *	1.236077071	0.959081971	0.911605999	0.838485712	0.954961348
**DHHC5**	1.025344342	0.945473864	1.216559083 *	1.013318952	0.791936107	0.923958511
**DHHC6**	1.018236721	1.03336611	1.057246266	1.093596115	1.080892738	1.069959107
**DHHC7**	0.90701589	1.057166156	1.030753647	1.391214277 *	1.276157037 *	1.269291019 *
**DHHC8**	1.028489044	1.098139884	0.969639483	1.279379166 *	1.309443602 *	1.401611743 *
**DHHC9**	1.030911399	0.989542024	1.191402677	1.049315467	0.870803772	0.925625144
**DHHC10**	1.04052826	0.955898406	1.179092429	1.030719429	1.082612436	0.941779505
**DHHC11**	1.048449108	1.025493987	1.191746378	1.032018325	0.799028932	0.970679961
**DHHC12**	1.02977489	1.035061735	1.241937271	1.252240752	0.936962825	1.191304031
**DHHC13**	1.012604219	1.135739789 *	1.075458329	1.382803155 *	1.327612762 *	1.162247512
**DHHC14**	1.11372248	0.935468441	0.977236983	1.560631752 *	1.334721037 *	1.266516085 *
**DHHC15**	0.988484105	1.083379301	1.201716091	1.004194734	0.910811999	0.943309239
**DHHC16**	1.05852722	1.118614437	0.961600161	1.270825703 *	1.232629386 *	1.032898324
**DHHC17**	1.164290762	1.266064823 *	1.188892722	1.138682969	1.18728256	1.271884081 *
**DHHC18**	0.958738591	1.057103531	1.268191413	1.044677662	0.957219103	1.023225855
**DHHC19**	1.061143632	1.016416393	1.184924658	1.030046952	0.822408029	0.906008507
**DHHC20**	1.022058933	1.03529984	1.207316875	0.979732069	0.824299588	0.92677786
**DHHC21**	1.39323177 *	1.048966849	0.962678734	1.943737964 *	1.676933126 *	1.139573616
**DHHC22**	1.026044765	1.061249666	1.154929576	1.004789142	0.822702454	0.9278397
**DHHC23**	1.037399478	1.009449177	1.332793291 *	1.000243923	0.802256917	1.018176706

## Data Availability

The original contributions presented in this study are included in the article. Further inquiries can be directed to the corresponding author.

## Supplementary Materials

The following supporting information can be downloaded at: https://www.mdpi.com/article/10.3390/biom15010067/s1, Figure S1: Gene expression of D*hhc*8 and D*hhc*14 was not different between non-stressed non-treated, imipramine- and DS-treated mice; Table S1: Sequences of the primers for GAPDH, D*hhc*3, D*hhc*7, D*hhc*8, D*hhc*13, D*hhc*14 and D*hhc*21.
