# Supplementary material for: Unveiling the Mechanisms of a Remission in Major Depressive Disorder (MDD)-like Syndrome: The Role of Hippocampal Palmitoyltransferase Expression and Stress Susceptibility
Source: Biomolecules. 2025 Jan 5;15(1):67. doi: 10.3390/biom15010067 (PMC11764023; doi:10.3390/biom15010067)
Supplement: Supplementary file 1 [file biomolecules-15-00067-s001.zip › biomolecules-3381387-supplementary.pdf]

Supplementary File

Gene expression of *Dhhc8* and *Dhhc14* in non-stressed non-treated, imipramine- and DS-treated groups of mice was examined in a separate assay. No significant group differences were found between non-stressed mice that were non-treated and non-stressed animals that received imipramine (7 mg/kg/day) or dicholine succinate (DS, 25 mg/kg/day) in the expression of *Dhhc8* (F=0.22, p=0.809, one-way ANOVA, post-hoc Tukey test) and *Dhhc14* (F=1.32, p=0.303, Supplementary Figure S1).

Supplementary Figure S1

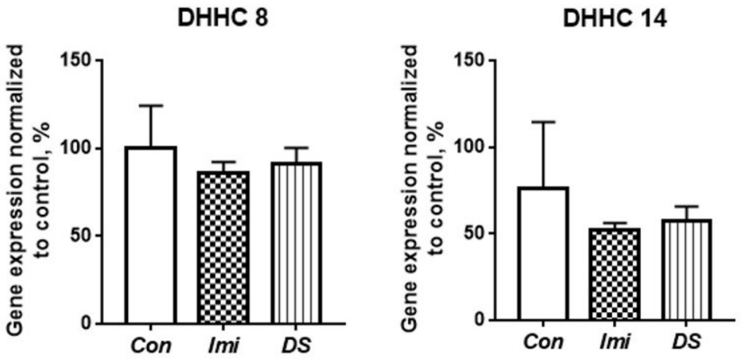

Supplementary Figure S1. Gene expression of D*hhc*8 and D*hhc*14 was not different between non-stressed non-treated, imipramine- and DS-treated mice. Con – control, Imi – imipramine, DS – dicholine succinate, all n=5. One-way ANOVA, Bars are mean ± SEM.

Supplementary Table S1

Name	Forward sequence	Reverse sequence
Gapdh	5′-ACCCCTTCATTGACCTCAACTACATG-3′	5′-CCTTCTCCATGGTGGTGAAGAC-3′
Dhhc3	5′-GGATCCATGATGCTTATCCCCACCCACCAC-3′	5′-TTCAGACCACATACTGGTACGGGTC-3′
Dhhc7	5′-GGATCCATGCAGCCATCAGGACACAGGCTCC-3′	5′-TTCACACTGAGAACTCCGGGCCGC-3′
Dhhc8	5′-ATCATGTGGCCCTGCAGCCCCTGCG-3′	5′-TTCACACCGAGATCTCGTAGGTGGTC-3′
Dhhc13	5′-AGATCTATGGGGCAGCCCTGGGCGGCTGGG-3′	5′-TTCAGGAGGCTGTGTGTCCCACATCTG-3′
Dhhc14	5′-CTTTATATTTGCATTCGTTATCACCCACG-3′	5′-TTCACACGGAGCTGAGCTTCACCAGG-3′
Dhhc21	5′-GGATCCATGGGTCTCCGGATTCACTTTGTTG-3′	5′-TTTAGACATGATTGGCAAAGTGGTAGG-3′

Supplementary Table S1. Sequences of the primers for GAPDH, D***hhc***3, D***hhc***7, D***hhc***8, D***hhc***13, D***hhc***14 and D***hhc***21.
